# Added predictive value of omics data: specific issues related to validation illustrated by two case studies

**DOI:** 10.1186/1471-2288-14-117

**Published:** 2014-10-28

**Authors:** Riccardo De Bin, Tobias Herold, Anne-Laure Boulesteix

**Affiliations:** Department of Medical Informatics, Biometry and Epidemiology, Ludwig-Maximilians-Universität, Marchioninistr. 15, 81377 München, Germany; Department of Internal Medicine 3, University Hospital Grosshadern, Ludwig-Maximilians-Universität, Marchioninistr. 15, 81377 München, Germany; Clinical Cooperative Group Leukemia, Helmholtz Center Munich for Environmental Health, Marchioninistr. 15, 81377 München, Germany

**Keywords:** Added predictive value, Omics score, Prediction model, Time-to-event data, Validation

## Abstract

**Background:**

In the last years, the importance of independent validation of the prediction ability of a new gene signature has been largely recognized. Recently, with the development of gene signatures which integrate rather than replace the clinical predictors in the prediction rule, the focus has been moved to the validation of the added predictive value of a gene signature, i.e. to the verification that the inclusion of the new gene signature in a prediction model is able to improve its prediction ability.

**Methods:**

The high-dimensional nature of the data from which a new signature is derived raises challenging issues and necessitates the modification of classical methods to adapt them to this framework. Here we show how to validate the added predictive value of a signature derived from high-dimensional data and critically discuss the impact of the choice of methods on the results.

**Results:**

The analysis of the added predictive value of two gene signatures developed in two recent studies on the survival of leukemia patients allows us to illustrate and empirically compare different validation techniques in the high-dimensional framework.

**Conclusions:**

The issues related to the high-dimensional nature of the omics predictors space affect the validation process. An analysis procedure based on repeated cross-validation is suggested.

## Background

In the last 15 years numerous signatures derived from high-dimensional omics data such as gene expression data have been suggested in the literature. A bitter disillusion followed the enthusiasm of the first years, as researchers realized that the predictive ability of most signatures failed to be validated when evaluated based on independent datasets. This issue is now widely recognized and validation is considered most important in omics-based prediction research by both quantitative scientists such as statisticians or bioinformaticians and medical doctors [1–6], see also topic 18 of the recently published checklist for the use of omics-based predictors in clinical trials [7].

A validation dataset can be generated by randomly splitting the available dataset into a training set and a validation set. This type of validation does not yield information on the potential performance of the signature on patients recruited in different places or at different times. The training and validation patients are drawn from the same population and are thus expected to be similar with respect to all features relevant to the outcome. In this case, validation can be seen as an approach to correct for all optimization procedures taking place while deriving the signature from the training data [8, 9]. External and temporal validations, in contrast, consider patients from a different place or recruited at a later time-point, respectively. They give information on the potential performance of the signature when applied to patients in clinical settings in the future. No matter how the validation dataset is chosen, the evaluation of prediction models using validation data is known to yield more pessimistic results than the evaluation performed on the training dataset using cross-validation or related techniques [6]. This is especially true when high-dimensional data are involved, since they are more affected by overfitting issues.

George [3] states that “the purpose of validation is not to see if the model under study is “correct” but to verify that it is useful, that it can be used as advertised, and that it is fit for purpose”. To verify that the model is useful, validation of the predictive ability of the omics model is not sufficient, as the clinical interest centers around the added value compared to previous existing models [10]. To verify that the new model is useful, one also needs to validate the added predictive value. This concept is not trivial from a methodological point of view and one may think of many different procedures for this purpose. While the problem of added predictive value has long been addressed in the literature for low-dimensional models, literature on added predictive value of signatures derived from high-dimensional data is scarce [11], although the high dimension of the predictor space adds substantial difficulties that have to be addressed by adapting classical methods.

In this paper we focus on this latter case, aiming to provide a better understanding of the process of validation of the added predictive value of a signature derived from high-dimensional data. We tackle this issue from an empirical perspective, using exemplary studies on the prediction of survival in leukemia patients which use high-dimensional gene expression data. Our goal is three-fold: (i) to demonstrate the use of different methods related to the validation of added predictive value, (ii) to show the impact of the choice of the method on the results, and (iii) to suggest an analysis approach based on our own experience and previous literature.

In order to better shed light on the methodological issues and the actual use of the validation methods, we take advantage of two leukemia datasets which are paradigm cases in biomedical practice. In particular, their relatively small effective sample size (number of events) is typical of this kind of study. It is worth noting, however, that a statistical comparison whose results could be generalizable needs a large number of studies [12] or convincing evidence from simulations, and therefore two examples would have been meant as illustrative even if they had had a larger effective sample size. Furthermore, these studies allow us to pursue our goals in two different situations: one, ideal from a statistical point of view, in which the omics data are gathered in the same way both in the training and in the validation sets, and one in which they are gathered with different techniques, making training and validation observations not directly comparable. In particular, in the first dataset we start from the work of Metzeler and colleagues [13], and we illustrate alternative approaches to study the added predictive value of their score, in addition to their performed validation strategy based on the p-value of a significance test in the Cox model. The second dataset, instead, allows us better insight into the approaches available in a situation in which a measurement error – in a broad sense including the use of different techniques to measure the gene expressions – makes the validation process more complicated. This is not uncommon in biomedical practice, especially since specific technologies, such as TaqMan Low Density Array, enable rapid validation of the differential expressions of a subset of relevant genes previously detected with a more labor-intensive technique [14]. Therefore, it is worth considering this situation from a methodological point of view. It is worth noting that the validation of the added predictive value concerns only the gene signature computed with data collected following the technique used in the validation set, not its version based on the training data. When the training and the validation data are not directly comparable, any analysis must be performed using only the information present in the validation set. In particular, a possible bad performance of the signature, in this case, would not mean an overall absence of added predictive value, but its lack of usefulness when constructed with data obtained with the latter technique.

We first present the considered leukemia datasets in the Data section in order to subsequently use them to illustrate the methods presented in the Methods section. These methods are compared empirically in the Results section. In order to improve transparency and facilitate the readability of our study, we summarize the description of the data used and the analyses performed in Tables 1 and 2, adapting the REMARK profile [15].

**Table 1:** **Acute myeloid leukemia: REMARK-like profile of the analysis performed on the dataset**

a) Patients, treatment and variables				
**Study and marker**		**Remarks**		
Marker		OS = 86-probe-set gene-expression signature		
Further variables		v1 = *age*, v2 = *sex*, v3 = *NMP1*, v4 = *FLT3-ITD*		
Reference		Metzeler et al. (2008)		
Source of the data		GEO (reference: GSE12417)		
**Patients**		**n**	**Remarks**	
Training set	Assessed for eligibility	163	**Disease:** acute myeloid leukemia	
			**Patient source:** German AML Cooperative Group 1999-2003	
	Excluded	0		
	Included	163	**Treatment:** following AMLCG-1999 trial	
			**Gene expression profiling:** Affymetrix HG-U133 A&B microarrays	
	With outcome events	105	**Overall survival:** death from any cause	
Validation set	Assessed for eligibility	79	**Disease:** acute myeloid leukemia	
			**Patient source:** German AML Cooperative Group 2004	
	Excluded	0		
	Included	79	**Treatment:** 62 following AMLCG-1999 trial 17 intensive chemotherapy outside the study	
			**Gene expression profiling:** Affymetrix HG-U133 plus 2.0 microarrays	
	With outcome events	33	**Overall survival:** death from any cause	
**Relevant differences between training and validation sets**				
Data source			Same research group, different time (see above)	
Follow-up time			Much shorter in the validation set (see text)	
Survival rate			Higher in the validation set (see Figure 2)	
**b) Statistical analyses of survival outcomes**				
**Analysis**	**n**	**e**	**Variables considered**	**Results/remarks**
*A: preliminary analysis (separately on training and validation sets)*				
A1: univariate	163	105	v1 to v4	Kaplan-Meier curves (Figure 1)
	79	33		
*B: evaluating clinical model and combined model on validation data (models fitted on training set, evaluated on validation set)*				
B1: overall prediction				Prediction error curves (Figure 5)
				Integrated Brier score (text)
	Training			Comparison of Kaplan-Meier curves for risk groups:
	163	105		- Medians as cutpoints (Figure 6),
B2: discriminative ability			OS, v1 to v4	- K-mean clustering (data not shown - see text)
				C-index (text)
	Validation			K-statistic (text)
B3: calibration	79	33		Kaplan-Meier curve vs average individual survival curves for risk groups (Figure 7)
				Calibration slope (text)
*C: Multivariate testing of the omics score in the validation data (only validation set involved)*				
C1: significance	79	33	OS, v1 to v4	Multivariate Cox model (Table 3)
*D: Comparison of the predictive accuracy of clinical and combined models through cross-validation in the validation data (only validation set involved)*				
D1: overall prediction	79	33	OS, v1 to v4	Prediction error curves based on repeated cross-validation (Figure 8)
				Prediction error curves based on repeated subsampling (data not shown - see text)
				Prediction error curves based on repeated bootstrap resampling (data not shown - see text)
				Integrated Brier score based on cross-validation (text)
*E: Subgroup analysis (E1-E3 based on training and validation sets, E4 and E5 only on validation set; for all, separate analysis for female and male population)*				
E1: overall prediction	Female		OS, v1 to v4	Prediction error curves (Figure 9)
E2: discriminative ability	t.: 88 54			C-index (text)
	v.: 46 16			K-statistic (text)
E3: calibration	Male			Calibration slope (text)
E4: significance	t.: 74 51			Multivariate Cox model (text)
E5: overall prediction	v.: 33 17			Prediction error curves based on cross-validation (Figure 10)

**Table 2:** **Chronic lymphocytic leukemia: REMARK-like profile of the analysis performed on the dataset**

a) Patients, treatment and variables				
**Study and marker**		**Remarks**		
Marker		OS = 8-probe-set gene-expression signature		
Further variables		v1 = *age*, v2 = *sex*, v3 = *FISH*, v4 = *IGVH*		
Reference		Herold et al. (2011)		
Source of the data		GEO (reference: GSE22762)		
**Patients**		**n**	**Remarks**	
	Assessed for eligibility	151	**Disease:** chronic lymphocytic leukemia	
			**Patient source:** Department of Internal Medicine III, University of Munich (2001 - 2005)	
Training set	Excluded	0		
	Included	151	**Criteria:** sample availability	
			**Gene expression profiling:** 44 Affymetrix HG-U133 A&B microarrays, 107 Affymetrix HG-U133 plus 2.0 microarrays	
	With outcome events	41	**Overall survival**	
	Assessed for eligibility	149	**Disease:** chronic lymphocytic leukemia	
			**Patient source:** Department of Internal Medicine III, University of Munich (2005 - 2007)	
Validation set	Excluded	18	Due to missing clinical information	
	Included	131	**Criteria:** sample availability	
			**Gene expression profiling:** 149 qRT-PCR (only selected genes)	
	With outcome events	40	**Overall survival**	
**Relevant differences between training and validation sets**				
Data source			Same institution, different time (see above)	
Measurement of gene expressions			Affymetrix HG-U133 vs. TaqMan LDA (see text)	
Survival rate			Lower in the validation set (see Figure 4)	
**b) Statistical analyses of survival outcomes**				
**Analysis**	**n**	**e**	**Variables considered**	**Results/remarks**
*F: preliminary analysis (separately on training and validation sets)*				
F1: univariate	151	41	v1 to v4	Kaplan-Meier curves (Figure 3)
	131	40		
*G: Multivariate testing of the omics score in the validation data (only validation set involved)*				
G1: significance	131	40	OS, v1 to v4	Multivariate Cox model (Table 5)
*H: Comparison of the predictive accuracy of clinical and combined models through cross-validation in the validation data (only validation set involved)*				
H1: Overall prediction	131	40	OS, v1 to v4	Prediction error curves based on cross-validation (Figure 11)
				Integrated Brier score based on cross-validation (text)

## Data

### Acute myeloid leukemia

The first dataset comes from a study conducted by Metzeler and colleagues [13] on patients with cytogenetically normal acute myeloid leukemia (AML). As one of the main results of the study, the authors suggest a signature based on the expression of 86 probe sets for predicting the event-free and overall survival time of the patients. In this paper we focus on the latter of the two outcomes, which is defined as the time interval between entering in the study and death. The signature was derived using the “supervised principal component” [16] technique, which in this study leads to a signature involving 86 probe sets. The supervised principal component technique consists of applying principal component analysis to the set of predictors mostly correlated with the outcome; in this specific case, the authors used the univariate Cox scores as a measure of correlation, and they selected those predictors with absolute Cox score greater than a specific threshold derived by a 10-fold cross-validation procedure.

The 86 probe set signature was derived using the omics information contained in a training set of 163 patients, with 105 events (patients deceased) and 58 right censored observations. The validation set included 79 patients, with 33 events and 46 right censored observations. Gene expression profiling was performed using Affymetrix HG-U133 A&B microarrays for the training set and Affymetrix HG-U133 plus 2.0 microarrays for the validation set. Both sets are available in the Gene Expression Omnibus (reference: GSE12417). Our starting point is the data as provided in the Web depository; see Table 1 for a brief description. For further details concerning specimen and assay issues, in accordance with the criteria developed by the US National Cancer Institute [7], we refer to the original paper [13]. We stress the importance, for clinical applicability of an omics-based predictor, of following the checklist provided by McShane and colleagues [7, 17].

**Figure 1:**
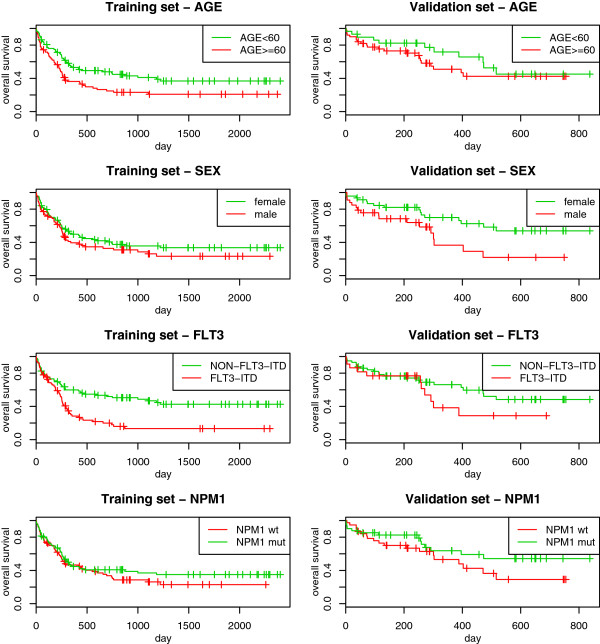
**AML: univariate Kaplan-Meier curves.** Acute myeloid leukemia: Kaplan-Meier estimation of the survival curves in subgroups based on *age* (first row), *sex* (second row), *FLT3-ITD* (third row) and *NPM1* (fourth row), computed in the training (first column) and in the validation (second column) sets.

**Figure 2:**
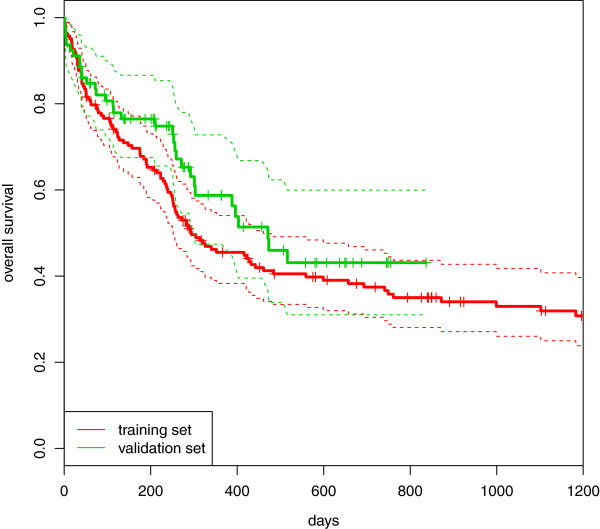
**AML: Kaplan-Meier curves.** Acute myeloid leukemia: comparison between the Kaplan-Meier estimation of the survival curves computed in the training (red line) and in the validation (green line) sets.

For both the training and validation sets, we also have information on some clinical predictors, namely *age*, *sex*, *FLT3-ITD* (internal tandem duplication of the fms-like tyrosine kinase 3) and *NPM1* (mutation in nucleophosmin 1). Here *age* is a continuous variable ranging from 17 to 83 years in the training set and from 18 to 85 in the validation set. The other three predictors are dichotomous (male/female, *FLT3-ITD*/*NON-FLT3-ITD* and *NMP1* mutated/wild type, respectively). For more information, we refer to the original paper [13]. To give an initial impression of the data, Figure 1 shows a first univariate graphical analysis of the clinical predictors based on the Kaplan-Maier curves, where the threshold used to dichotomize the predictor *age* (60 years) is established in the medical literature [18]. It can be immediately seen that there is a large difference in the follow-up times: in the training set, it ranges from 0 to 2399 days (median 1251, computed by inverse Kaplan-Meier estimate); in the validation set, from 1 to 837 days (median 415). The events in the training set mainly occur in the first 800 days, and therefore the non-overlapping time is not highly informative; in contrast in the validation set there are no events after 1.5 years (547.5 days), which suggests the existence of a non-negligible difference between the two sets. From the analysis of the Kaplan-Meier curves, we can also see that the effect of the predictor *FLT3-ITD* seems to vary over time (this issue is more visible in the validation set, where *FLT3-ITD* seems to have no effect in the first 250 days, while for the training set it seems to have no effect only in the first 150 days). All the other predictors, however, seem to have regular behavior, and in the multivariable Cox model that includes all clinical predictors, the proportional hazards assumption is acceptable. Finally, the two sets differ slightly in terms of survival rate. As can be seen in Figure 2, the patients in the validation set have a lower mortality than those in the training set (for graphical clarity, here the Kaplan-Meier curve for the training set is cut at 1250 days, after the last event).

### Chronic lymphocytic leukemia

The second dataset comes from a study conducted by Herold and colleagues [19] on patients with chronic lymphocytic leukemia (CLL). The main goal of this study is also to provide a signature based on gene expression which can help to predict time-to-event outcomes, namely the time to treatment and the overall survival time. We again focus on the overall survival, as the authors did. The signature developed in this study is based on the expression of eight genes and was obtained using the “supervised principal component” technique, similarly to the previous study. In this study, however, the selection of the relevant gene expression predictors is more complex. The univariate Cox regressions measuring the strength of the association between survival time and each of the candidate predictors are not simply conducted based on the whole dataset like in the previous study, but are instead repeated in 5000 randomly drawn bootstrap samples. In each of these samples, the association between each predictor and the outcome was computed, and the predictors with a significant association were selected. The 17 genes most frequently selected across the 5000 bootstrap replications were considered in a further step, which was necessary to discard highly correlated genes. The expressions of the 8 genes surviving this further selection were finally used to construct the prognostic signature. The use of a procedure based on bootstrap sampling is motivated by the necessity of increasing the stability and potentially reducing the influence of outliers [20].

For this study, there was also a training set that was used to derive the signature, and an independent validation set that was used to evaluate its accuracy. The former contains clinical and omics information on 151 patients, with 41 events and 110 right censored observations. Among the 149 patients from the validation set, 18 were discarded due to missing values, resulting in a sample size of 131, with 40 events and 91 censored observations. The gene expression data are available in the Gene Expression Omnibus with reference number GSE22762. Further information about the omics data, as provided in the Web depository, is collected in Table 2. For this dataset as well, we refer the reader to the original paper [19] for the additional details on the preliminary steps of data collection/preparation (and their compliance with the US National Cancer Institute’s criteria for the clinical applicability of an omics-based predictor [7]).

The peculiarity of this study is that the gene expressions were collected using a different technique for the training set than for the validation set. The training set gene expressions were measured using Affymetrix HG-U133 (44 Affymetrix HG-U133 A&B, 107 Affymetrix HG-U133 plus 2.0), while for the validation patients a low-throughput technique (TaqMan Low Density Array, LDA) was used to measure only those genes involved in the signature. The validation procedures, therefore, are restricted to use only the validation data and cannot take into consideration the training set.

The considered clinical predictors were *age* (considered continuous as in the previous study), *sex*, fluorescent in situ hybridization (*FISH*) and immunoglobulin variable region (*IGVH*) mutation status. *FISH* and *IGVH* are two widely used predictors in CLL studies [21]. The former is an index based on a hierarchical model proposed by Döhner and colleagues [18] that includes the possible deletion or duplication of some chromosomal regions (17p13, 11q22-23, 13q14, 12q13), and has 5 modalities (0 = deletion of 13q14 only, 1 = deletion of 11q22-23 but no deletion of 17p13, 2 = deletion of 17p13, 3 = trisomy 12q13 but no deletion of 17p13 or 11q22-23, 4 = no previously mentioned chromosomal aberration), while the latter indicates whether *IGVH* is mutated or not.

**Figure 3:**
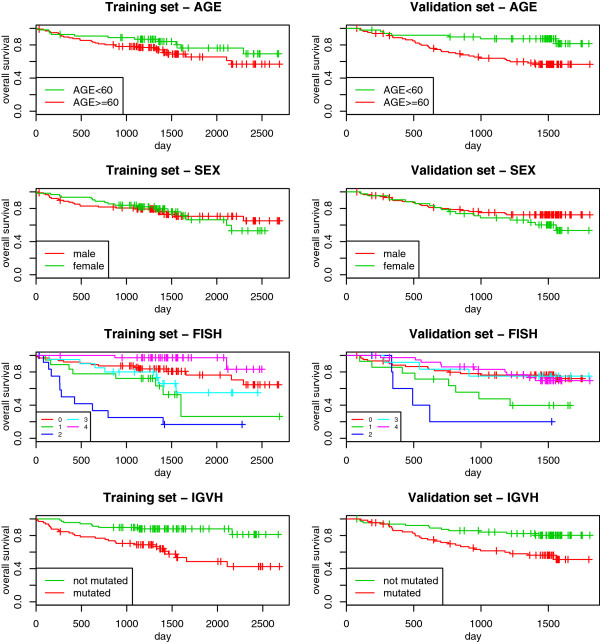
**CLL: univariate Kaplan-Meier curves.** Chronic lymphocytic leukemia: Kaplan-Meier estimation of the survival curves in subgroups based on *age* (first row), *sex* (second row), *FISH* (third row) and *IGVH* (fourth row), computed in the training (first column) and in the validation (second column) sets. The values of *FISH* (third row) represent: 0 = deletion of 13q14 only; 1 = deletion of 11q22-23 but no deletion of 17p13; 2 = deletion of 17p13, 3 = trisomy 12q13 but no deletion of 17p13 or 11q22-23, 4 = no previously mentioned chromosomal aberration.

**Figure 4:**
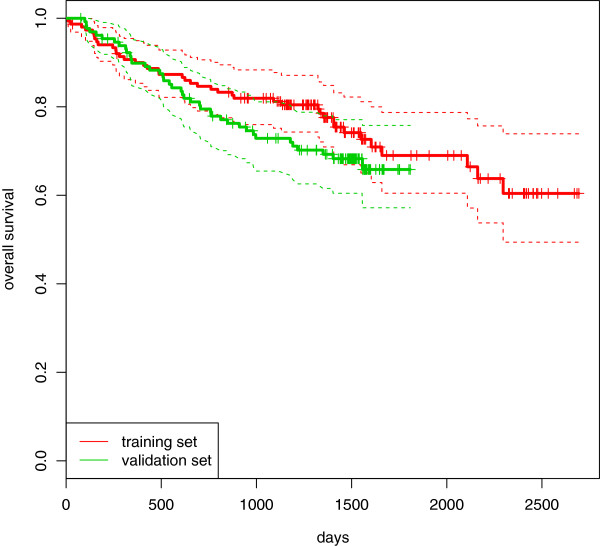
**CCL: Kaplan-Meier curves.** Chronic lymphocytic leukemia: comparison between the Kaplan-Meier estimation of the survival curves computed in the training (red line) and in the validation (green line) sets.

Here again we present a preliminary overview of the univariate effect of the clinical predictors via the Kaplan-Meier curves. The results are reported in Figure 3. We can see that both *FISH* and *IGVH* are able to separate patients with high risk and patients with low risk well. In particular, the difference between patients with *FISH* = 2 (patients with “deletion 17p13”) and the others is obvious. This group is characterized by a small sample size and very high risk of death. For this study there is a smaller difference in terms of follow-up time between the training and the validation sets: in the former, it ranges from 11 to 2694 days (median computed via reverse Kaplan-Meier curve equal to 1499); in the latter, from 77 to 1808 days (median 1516). Further, there is a small difference between the two sets in terms of survival rate. In Figure 4, we can see that the Kaplan-Meier curve computed for the validation set is below the one computed for the training set.

## Methods

### Scores

The term “signature” usually refers to a score synthesizing several omics markers that is supposed to be related to the patient’s disease status or outcome. In this paper, we prefer the term “omics score”, which better emphasizes how the score is constructed and clearly outlines its quantitative character. An omics score is typically derived by applying an algorithm to a training set. It can either involve all the features present in the dataset or a subset of them. For example, in the CLL study (see Data section for more details), the authors selected (a subset of) eight genes and defined their omics score as the first principal component:


where the abbreviation OS stands for omics score and the other abbreviations are the names of the involved genes. This score is linear, but in general scores may also show a more complex structure. In some cases they do not even have a simple closed form, for example when they are derived using machine learning tools like random forests.

### Strategies

No matter with which algorithm the omics score was derived from the training data, its usefulness as a predictor for prognosis purposes has to be evaluated using a set of patients that have not been considered until now: the validation data. We now focus on this part of the analysis, with special emphasis on the question of the added predictive value given other well-established clinical predictors. The underlying idea is that the new omics score is relevant for clinical practice only if it improves the prediction accuracy [22] that one would obtain from existing predictors. An exception where the omics score may be useful even if it does not improve prediction accuracy is when it is, say, cheaper or faster to measure. We assume that this is not the case in most applications and that the question of the *added* predictive value is an important issue.

Here we consider the following situation: we have at our disposal the clinical data (predictors ) and the omics data (predictors ) for both the training and the validation sets. Furthermore, we know how the omics score can be calculated from the omics data. In the case of linear scores like those suggested in the two considered leukemia studies, it means that we know the coefficients and the name of each involved gene, either from a table included in a paper or from a software object. In the rest of this paper, the function used to calculate the omics score from the omics predictors  will be denoted by , where the hat and the superscript  indicate that this function was estimated based on the training set.

**A. Evaluating the clinical model and the combined model on validation data.** The most direct approach to the validation of the added predictive value of an omics score consists of (i) fitting two models to the training data: one involving clinical predictors only and one combining clinical predictors and the omics score of interest, and (ii) evaluating their prediction accuracy on the validation set. The added predictive value can then be considered validated if the prediction accuracy of the combined score (i.e., the score involving both the clinical predictors and the omics score) is superior to the prediction accuracy of the clinical score (i.e., the one based only on clinical predictors). This general approach has to be further specified with respect to

1. the procedure used to derive a combined prediction score;
2. the evaluation scheme used to compare the prediction accuracy of the clinical and combined prediction scores on the validation set.

Regarding issue 1), a natural approach consists of simply fitting a multivariate Cox model with the clinical predictors and the omics score as predictors to the training data. The resulting linear score can then be regarded as a combined score, since it involves both clinical predictors and the omics score. More precisely, the model
1

is fit by maximization of the partial likelihood on the training set, yielding the estimates  (for ) and , where the exponent  stands for the training dataset that is used for fitting. In model (1), the omics score OS is given as OS. The clinical model
2

is computed in the same way, without taking into account the omics information.

Regarding issue 2), we need to specify how we measure the prediction accuracy of the prognostic rules based on the clinical and the combined prediction scores. This involves a graphical or numerical investigation of their *discriminative ability* and *calibration*, either separately or simultaneously. We will focus later on this issue 2 in a dedicated section, “Evaluation criteria”. In the meantime, we want to stress that, within this strategy (strategy A), the measure of the prediction accuracy is computed in the validation set. There is a major issue related to this approach: the omics score, fitted to the training data, tends to (strongly) overfit these data and to consequently dominate the clinical predictors. This is because the training set  is used twice: first for the estimation of  and then for the estimation of . This issue will be discussed further when examining the application to our two exemplary datasets.

**B. Multivariate testing of the omics score in the validation data.** To address this overfitting issue, model (1) can also be fitted on the validation data, yielding the estimates  (for ) and  for the clinical predictors  and the omics score OS, respectively. Here the exponent  stresses the fact that the estimates are computed using the validation data. By fitting the model on the validation data, we do not face the overfitting issues mentioned above, because different sets are used to derive OS and to fit the coefficients of model (1). In this approach the clinical predictors of the training set are not used.

A test can then be performed to test the null-hypothesis *β*_∗_=0, for instance a score test, a Wald test or a likelihood ratio test. The p-value can be used as a simple and familiar measure of association between the score and the outcome. However, the p-value is more related to the explained variability than to the prediction error, and a small p-value can also be found if the omics score hardly adds anything to the predictive value [11]. Therefore, the use of the p-values for the validation of the additional predictive value of an omics score is not sensible. For example, the p-value gets smaller simply by increasing the sample size, even if the predictive ability of the model does not change [11].

**C. Comparison of the predictive accuracy of the models with and without omics score through cross-validation in the validation data.** To focus on predictive ability, one option consists of evaluating the combined model (1) and the model based on clinical data only (2) through cross-validation (or a related procedure) on the validation set. The main reason to perform this procedure is to avoid the overfitting issues related to the aforementioned double use of the training data for variable selection and parameter estimation. The cross-validation procedure mimics the ideal situation in which three sets are available: one to construct the omics score, one to estimate the parameters and one to test the model. This is performed by splitting the validation set into *k* subsets: in each of the *k* iterations, the outcome of the *k*-th fold (“test set”) is predicted using both the clinical and the combined models fitted in the remaining *k*-1 folds (“learning set”) in turn. Comparing these predictions with the actual values of the outcome present in the *k*-th fold, we can compute a measure of prediction accuracy. As already stated for strategy A, the prediction accuracy of the prognostic rules based on the clinical and the combined prediction scores can be measured in terms of discriminative ability, calibration, or these two properties simultaneously. The details are explained in the dedicated section. Since in each cross-validation step parameter estimation and measurement of the prediction accuracy are performed in independent sets, we do not face overfitting issues. The averages of the results (in terms of prediction accuracy) obtained in the *k* iterations for the two models allow the assessment of the added predictive value of the omics score.

Note that for this approach standard multivariate Cox regression may be replaced by any other prediction method if appropriate, for example a method which deals better with the collinearity of the clinical predictors  and the omics score.

**D. Subgroup analysis.** Subgroup analyses may be helpful in the context of added predictive value for different reasons. Firstly, biological reasoning may be available. If there are few existing predictors, examining the performance of the omics score in all possible subgroups defined by the existing predictors is a direct approach to determine its *added* predictive value, i.e. whether it can discriminate between patients when existing predictors cannot (since they have the same values for all predictors). Secondly, even if there are too many combinations of existing predictors to apply this direct approach, applying the methods described in the above sections to subgroups may yield interesting results, for instance that the omics score has more added predictive value in a particular subgroup. The most important drawbacks of such subgroup analyses are related to sample size (each subgroup being smaller than the whole dataset) and multiple testing issues (if several subgroups are investigated in turn). Care is required in assessing the value of subgroup analyses.

### Evaluation criteria

In the description of the different strategies, we have seen that a relevant aspect of validating the added predictive value of an omics score is how to measure the prediction accuracy of a prognostic rule. As we stated above, this can be done by investigating, either separately or combined, the discriminative ability and the calibration. Specifically, the former describes the ability to discriminate between observations with and without the outcome, or, in the case of continuous outcome, correctly ranking their values: in the case of survival data, for example, predicting which observations have the higher risk. Since in this paper we focus on survival analysis, we refer only to those methods that handle time-to-event data. This is true also for the calibration, which, in this context, can be seen as a measure describing the agreement between the predicted and the actual survival times.

**Discriminative ability:** In the context of survival curves, the discriminative ability is, in principle, reflected by the distance between the survival curves for individuals or groups [23]. Therefore, a graphical comparison between the Kaplan-Meier curves can be used to assess this property: the best rule, indeed, is the one which leads to the most separated curves. In practice, we can split the observations into two groups, assembled considering the estimates of the linear predictors  and , for example, using their medians as cutpoints. In this way, we define a low- and a high-risk group for both cases (using *η*_*comb*_ and *η*_*clin*_), and we can plot the resulting four Kaplan-Meier curves. If the two curves related to the groups which are derived using  are much more separated than those related to the groups derived using , then we can assert the presence of added predictive value. In principle, more prognostic groups can be constructed, reflecting a division more meaningful from a medical point of view. Nevertheless, for the illustration purpose of this graphic, the two-group split is sufficient. In the same vein, the choice of the cutpoint is also not relevant, and we would expect similar results with different (reasonable) cutpoints.

Numerical criteria, instead, can be based on the estimation of the concordance probability or on the prognostic separation of the survival curves. The most popular index which exploits the former idea is probably the C-index [24]. It consists of computing the proportion of all the “usable” pairs of patients for which the difference between the predicted outcomes for the pairs and the difference between the true outcomes for the pairs have the same sign. Here “usable” means that censoring does not prevent the ordering of them. This limitation shows the dependence of this index on the censoring scheme, which may compromise its performance. To cope with this issue, in this paper we use the version of the C-index described in Gerds and colleagues [25]. Moreover, for the same reason, we also consider the alternative index proposed by Gönen & Heller [26], which relies on the proportional hazards assumption and is applicable when a Cox model is used. For both indexes, the highest value denotes the best rule (on a scale from 0 to 1).

**Calibration:** The calibration can also be evaluated graphically. A simple method consists of comparing the Kaplan-Meier curve (observed survival function) computed in the validation set with the average of the predicted survival curves of all the observations of the validation sample [23]. The closer the predicted curve is to the Kaplan-Meier curve, the better calibration the prognostic rule has. Under the proportional hazards assumption, a numeric result can be obtained via the “calibration slope”. This particular approach consists of fitting a Cox model with the prognostic score as the only predictor. Good calibration leads to an estimate of the regression coefficient being close to 1. It is worth pointing out that this procedure focuses on the calibration aspect and does not constitute itself, as sometimes claimed in the literature, a validation of the prediction model [23]. Calibration is often considered less important than discriminative ability, because a recalibration procedure can be applied whenever appropriate.

**Overall performance:** a measure of the overall performance of a prognostic rule should incorporate both discrimination and calibration. The integrated Brier score [27, 28] is such a measure. It summarizes in a single index the time-dependent information provided by the Brier score [29] (which measures the prediction error at a specific time *t*), by integrating it over the time. The best prediction rule is the one which leads to the smallest value for the integrated Brier score. The Brier score can also be plotted as a function of time to provide the prediction error curve, which can be used to graphically evaluate the prediction ability of the model: the lower the curve, the better the prediction rule is. The integrated Brier score corresponds to the area under this curve.

We note that, in order to compute these measures, different levels of information from the training set are needed [23]. For example, the baseline hazard function is necessary to assess calibration, while it is not needed to evaluate the discriminative ability via Kaplan-Meier curves.

The literature provides several other measures for the evaluation of the prediction ability of a model for survival outcomes [23, 28]. They can be either specifically developed in the survival analysis framework, such as the Royston-Sauerbrei D-measure [30] or Zheng et al. [31]’s positive prediction value, or adapted from different context, for example from classification problems. Examples of the latter kind of measures are the net reclassification index [10, 32, 33] and decision curve analysis [34, 35]. Finally, different variants of model-based *R*^2^-type coefficients of explained variation are also commonly used in the literature for different regression models, including models for survival outcomes [36]. All these alternative measures can be easily computed within the described strategies instead of the measures considered in this paper, providing different insights into the added predictive value of the omics score, but without changing the general principles. We summarize the characteristics of the measures considered in our paper in Table 3.

**Table 3:** **Characteristics of the measures implemented to evaluate the prediction ability of a model**

Aspect	Measure	Characteristics
Discriminative ability	Kaplan-Meier curves for risk groups	Better with greater distance between the Kaplan-Meier curves for the low- and high risk groups
	C-index	Estimates the concordance probability, i.e. the probability that the score correctly orders two patients with respect to their survival time; higher values correspond to better prediction
	K-statistic	Alternative to the C-index; works only under the proportional hazards assumption
Calibration	Survival curves	Compares the observed survival function with the average predicted curve
	Calibration slope	Computes the regression coeffcient of the prognostic score as unique predictor; the best values are those close to 1; related to overfitting issues
Overall prediction	Prediction error curves	Presents the Brier score versus time; the closer the curves are to the X-axis, the better the prediction
	Integrated Brier score	Computes the area under the prediction error curves; the smaller is the value, the better the prediction

## Results

### Acute myeloid leukemia

In this subsection we illustrate the application of different methods and their impact on the results by using the acute myeloid leukemia dataset. For a summary of the analyses performed, we refer to the profile provided in Table 1.

**A. Evaluating the clinical model and the combined model on validation data.** We have seen that the easiest way to derive a prediction combined score is to fit a multivariate Cox model which includes as covariates the clinical predictors and the omics score. The added predictive value of the latter is then validated by looking at the prediction properties (calibration, discrimination, overall performance) of this model compared to the model fitted using only the clinical predictors. For the AML dataset, therefore, we compare the combined model (see Table 4) with the clinical model (i.e., the model without the omics score).

**Figure 5:**
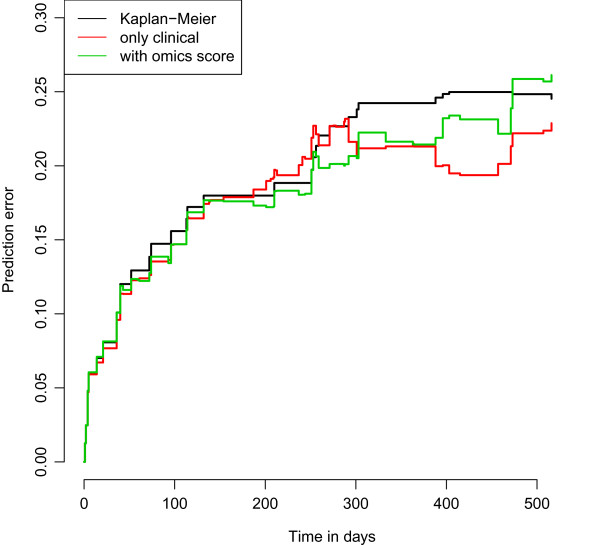
**AML: prediction error curves.** Acute myeloid leukemia: prediction error curves based on the Bier score computed in the validation set for the null (black line), the clinical (red line) and the combined (green line) models fitted on the training data.

While estimates from these models come from the training set, the prediction properties must be evaluated in the validation set. Starting by gaining an overall view of their predictive ability, we consider the Brier score, both by investigating graphically the prediction error curves representing its value versus time (Figure 5) and by measuring the area under these curves, commonly called the integrated Brier score. Since for late time-points the error estimates (Brier scores) are based on a small number of observations (generally with few/no events) and are therefore unreliable, the researcher may prefer to evaluate Brier score-based quantities up to a specific time, which would ideally have a clear clinical meaning. In this case, since we do not have any time value highly relevant from a clinical point of view, we choose to compute the integrated Brier score up to 1.5 years, following the graphical investigation of the Kaplan-Meier curves performed in the Data section. The values of the integrated Brier score are 0.201, 0.181 and 0.190 for the null, the clinical and the combined models, respectively, and, therefore, we cannot validate the added predictive value of the omics score. The graphical investigation of the prediction error curves in Figure 5 confirms this point: after an initial time period of around 300 days in which the three lines are indistinguishable (i.e., the prediction models do not provide any information), the red (clinical model) and the green (combined model) fall below the black (null model), showing that there is an advantage in using a prediciton model. Nevertheless, there is no evidence of a better performance of the combined model compared to the clinical model (the green line is not constantly below the red line).

**Figure 6:**
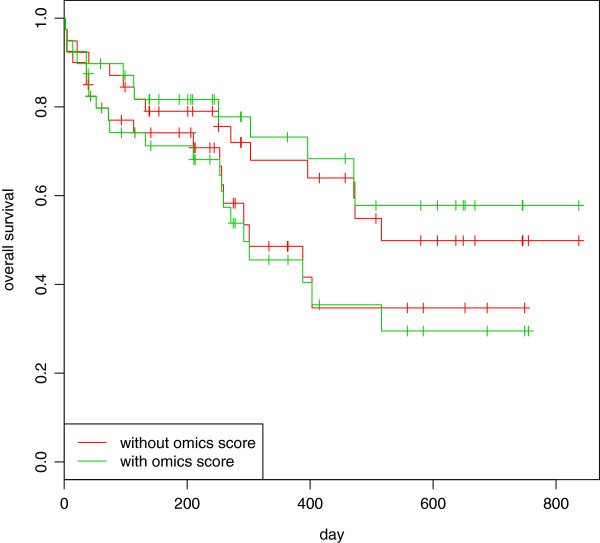
**AML: Kaplan-Meier curves for low and high-risk groups.** Acute myeloid leukemia: Kaplan-Meier curves computed in the validation set for risks groups based on the clinical (red) and the combined (green) scores derived in the training set: the curves below represent the survival curves for observations belonging to the high risk group, the two above the low risk group.

If we consider calibration and discriminative ability separately, we can see that the main issues are related to the former. The discriminative ability of the combined model, indeed, is slightly better both according to the C-index (0.631 versus 0.605 for the clinical model) and to the K-statistic (0.674 versus 0.653). The difference, however, is definitely not large, and the values themselves are small (the C-index and the K-statistic range from 0.500, which corresponds to a complete random situation, to 1, which indicates perfect concordance). We can draw the same conclusion from graphical inspection of Figure 6: the graphic shows the Kaplan-Meier curves for the low- and the high-risk groups (defined using the median score as a cutpoint) derived using the combined (green line) and the clinical (red line) models. The green lines are slightly more separated than the red ones, showing a little improvement in discrimination. We also tried to define low- and high-risk groups using a K-means clustering procedure (2-means), obtaining very similar results (here not shown).

**Table 4:** **Acute myeloid leukemia: estimates of the log-hazard in a multivariate Cox model fitted on the validation data, with the standard deviations and the p-values related to the hypothesis of nullity of the coefficients (simple null hypothesis)**

Variable	Coeff	Sd(coeff)	P-value
*Omics score*	0.523	0.243	0.0312
*Age* (continuous)	0.022	0.015	0.1340
*Sex* (male)	0.643	0.404	0.1114
*FLT3-ITD*	0.436	0.440	0.3220
*NPM1* (mutated)	-0.377	0.404	0.3497

**Figure 7:**
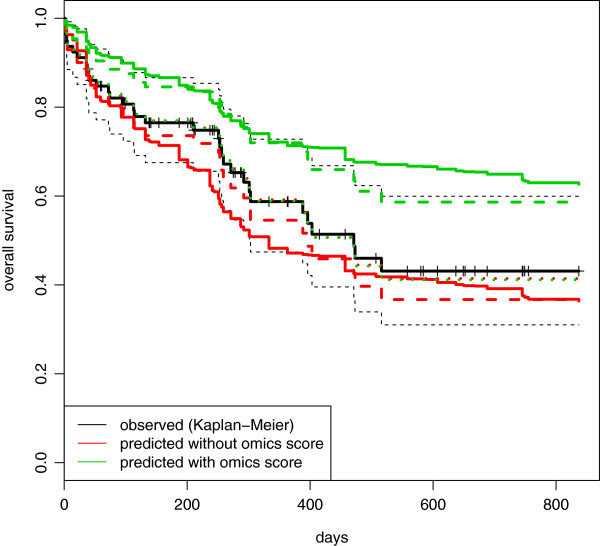
**AML: comparison between observed and average predicted survival curves in the validation set.** Acute myeloid leukemia: comparison between the observed survival curve (Kaplan-Meier, black line) and the average predictive survival curves computed in the validation set using the clinical (red line) and combined (green line) models fitted on the training data. Continuous lines represent the average predictive survival curves computed interpolating the baseline survival curve derived in the training set. Dashed lines represent the same curves computed using an estimation of the baseline survival curve derived in the validation set. For the dotted curves, the estimates of the regression coefficients are shrunk toward 0.

A different result is obtained when considering calibration. Figure 7 displays the graphical comparison between the Kaplan-Meier curve, i.e., the observed survival curve (continuous black line), and the average predicted survival curves (continuous line) of the subjects in the validation set, for both the clinical (red) and combined (green) models. Both predicted curves are relatively far from the observed one. This poor calibration is partly due to the difference between the two sets, which leads to different estimates of the baseline survival function (calibration-in-the-large): in order to show the effect of this difference, we have reported in a dashed line the average survival curves predicted using the baseline survival function computed in the validation data (please note that this is done to interpret the graphic, for validation purposes only the continuous lines are relevant). We can see that with this “correction”, the average survival curves becomes slightly closer to the observed one. The other aspect that we should consider is the calibration slope: being directly related to the linear predictors, it is of high interest in terms of validation of the added predictive value of the omics score. In order to focus on this aspect, we obtain a numerical result by estimating the regression coefficients of the clinical and of the combined score when used as a predictor in a Cox model. Since the intercept is absorbed in the baseline hazard, indeed, this procedure does not take into account the calibration-in-the-large [37]. The values obtained for the calibration slope confirm the impressions of the graphical investigation: the estimates of the regression coefficient using the clinical score and the combined score are 0.900 (sd =0.314) and 0.888 (sd =0.245), respectively. There is a slight worsening when considering the omics score, and both values are relatively far from the ideal case, in this case a coefficient equal to 1.

**Possible sources of overfitting.** As stated by Steyerberg et al. (2010) [28], calibration-in-the-large and calibration slope issues are common in the validation process, and they reflect the overfitting problem [38] that we mentioned previously in the Methods section. With particular regard to calibration slope, the overfitting issue can be related to the need for the shrinkage of regression coefficients [28, 39, 40]. If we go back to Figure 7 and shrink the regression coefficients toward 0, we can see that, in this way, we obtain good calibration (dotted lines, almost indistinguishable from the black one). In the clinical model, the shrinkage is performed by applying a factor of 0.92 to all four regression coefficients: the small amount of shrinkage necessary to move the average predicted curve close to the observed one reveals the relatively small effect of the overfitting issue in a model constructed with low-dimensional predictors. In order to obtain the same results with the combined model, instead, we applied a relatively large shrinkage factor, 0.5, to the regression coefficient related to the omics score (and, therefore, leaving those related to the clinical predictors unchanged). This reflects the typical situation of a model containing a predictor derived from high-dimensional data: since this predictor (omics score) has been constructed (variable selection and weight estimation) and its regression coefficient estimated in the same set (the training set), the overfitting issue largely affects the combined model. The fact that we need to apply the shrinkage factor only to the regression coefficient of the omics score, moreover, is a clear signal of how much the omics score, inasmuch derived from high-dimensional data, dominates the clinical predictors. This may explain the large distance between the red and the green (continuous) lines in Figure 7. As a result, the effect of the (possibly overfitting) omics score may turn out to hide the contribution of the clinical predictors when estimated on the same training set, in a way that in the validation step we in fact mostly evaluate the predictive value of the omics score. The fact that the problem of overfitting largely affects the calibration of the models, moreover, may influence the analyses based on a direct computation of the Brier score (strategy A), and a more refined approach (strategy C) may be required.

To highlight the overfitting problem, we re-estimated the regression coefficients of the combined model using the validation set. Table 5 shows the estimated log-hazard ratios of all considered predictors based on the training set (first column) and the validation set (second column). It can be seen that the log-hazard ratios of the predictors age, *FLT3-ITD* and *NPM1* are not noticeably different, while the value of the log-hazard ratio of the omics score decreases substantially from the training set – where overfitting is plausible – to the validation set. This confirms our suspicions and strengthens the idea that, if the effect of the omics score is to be assessed through a multivariate model, this model cannot be fitted on the same set used for the construction of the score (training set), but instead needs to be fitted on an independent dataset. Obviously, if we use the validation set for this purpose, i.e., as in van Houweliengen’s definition [41], to update the model, we need a third set for the validation. We have seen that this idea motivates strategy C.

**Table 5:** **Acute myeloid leukemia: differences in the estimates of the log-hazard ratio when the combined model is fitted on the training (first column) or on the validation (second column) data**

	Log-hazard ratios	
Variable	Training	Validation
*Omics score*	0.642 (0.172)	0.523 (0.243)
*Age* (continuous)	0.021 (0.008)	0.022 (0.015)
*Sex* (male)	-0.024 (0.208)	0.643 (0.404)
*FLT3-ITD*	0.448 (0.253)	0.436 (0.440)
*NPM1* (mutated)	-0.370 (0.215)	-0.377 (0.404)

Standard deviations are reported between brackets.

**B. Multivariate testing of the omics score in the validation data.** The combined multivariate model previously fitted on the training set can be further used to derive the p-value corresponding to the null-hypothesis that the coefficient of the omics score is zero, by estimating its regression coefficients on the validation set. The results are reported in Table 4, and are in line with those presented in the original paper [13]. More precisely, the authors used as clinical predictors only *age*, *FLT3-ITD* and *NPM1*, while here we also consider *sex*. Nevertheless, the effect of *sex* being weak (with a p-value of 0.111), the p-value of the score that we are interested in is hardly affected by this additional predictor (here p-value = 0.031, in the original paper, 0.037). Since these values are in a borderline area between the most commonly used significance levels of 0.01 and 0.05, we cannot clearly confirm the added predictive value of the omics score. Most importantly, this significance testing approach within the multivariate model does not provide any information on prediction accuracy, an aspect that is considered in the next section.

**Figure 8:**
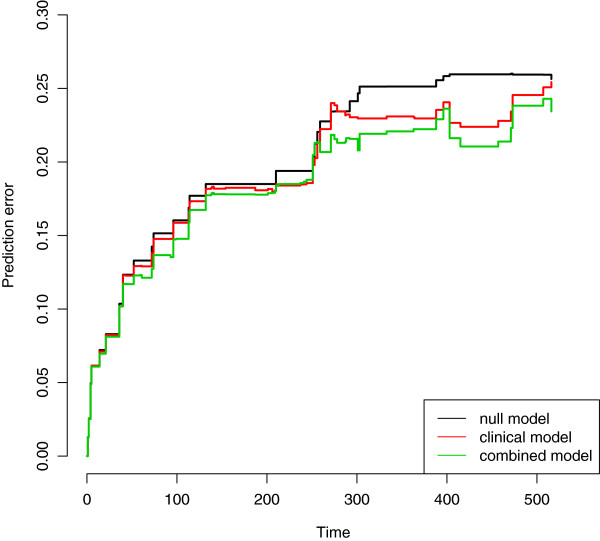
**AML: prediction error curves based on 10-fold cross-validation.** Acute myeloid leukemia: prediction error curves based on Brier score computed via 10-fold cross-validation (100 replications). The null (black line), the clinical (red line) and the combined (green line) models are considered. Only the validation set is used.

**C. Comparison of the predictive accuracy of the models with and without omics score through cross-validation in the validation data.** The combined model fitted on the validation set in the last subsection cannot be evaluated using the validation set again: the same set, indeed, cannot be used both to update and to validate the model. Since a third set is rarely available, an option is to evaluate this model based on a cross-validation approach (10-fold CV in this paper) as described in the Methods section, and to ultimately compare its performance to the performance of the model including clinical predictors only. Since the results of cross-validation usually depend highly on the chosen random partition of the data [42, 43], we repeat cross-validation 100 times for different random partitions and finally average the results over these repetitions. The results are reported in terms of Brier score via the prediction error curves in Figure 8. Although the clinical and the combined models have very similar behaviors, we can see a little improvement by including the omics score in the prediction model. This is probably not sufficient to clearly validate its added predictive value (thus agreeing with the borderline result obtained with the previous approach), but it confirms the influence of the overfitting issue: as we saw in Table 5, the regression coefficient for the omics score fitted in the training set seems to be too dependent on the training data, leading to prediction errors (Figure 5) for the combined model bigger than for the clinical one. When we fit the models on the validation data, as in this case, the problem disappears, and the combined model performs better than the clinical one (Figure 8). The values of the integrated Brier score computed for the different models (all up to 1.5 years) confirm these results: for the null model it is 0.208, 0.191 for the clinical and 0.188 for the combined. It is worth noting, however, that in the first 300 days the behaviors of the three curves are similar, strengthening the considerations stated for approach A.

Note that other resampling techniques for accuracy estimation might be used in place of 10-fold cross-validation. The respective advantages and pitfalls of these techniques have been the topic of a large body of literature [44, 45]. As a sensitivity analysis, we also performed our analysis using a 3-fold cross-validation procedure (repeated 100 times): the results, however, are very similar (data not shown). Please note that the repeated cross-validation is very similar to the repeated subsampling procedure, which has often been used in the context of high-dimensional data analysis [46–48]. The latter considers at each iteration only one of the *k* cross-validation splits into learning and test sets. For a large number of subsampling iterations or a large number of cross-validation repetitions, respectively, both procedures are known to yield similar results [49], which was corroborated by our preliminary analyses (data not shown). Another alternative is the bootstrap: in each bootstrap iteration, the models can be fitted on a bootstrap sample (i.e., a sample randomly drawn with replacement from the validation set) and then evaluated using those observations that are not included in the bootstrap sample. Using the “0.632+” version of bootstrap introduced by Efron and Tibshirani [50], based on 1000 bootstrap replications, we obtain results very similar to those obtained by the aforementioned techniques (data not shown).

**D. Subgroup analysis (male and female populations separately).** For this acute myeloid leukemia dataset, therefore, all the approaches seem to agree on the scarce improvement of including the omics score in the model in term of prediction ability. One aspect that remains to be investigated is the peculiar behavior of the predictor *sex*, which yields substantially different regression coefficient estimates in the training and validation sets (Table 5). Although the relevance of this predictor in the analysis is not obvious (it would have certainly been discarded by a variable selection procedure in the training set, the p-value related to a significance test in the validation set being 0.1114; see Table 4), it is the best candidate to use as a splitting criterion in order to illustrate the subgroup analysis described in the Methods section, and to highlight possible issues related to this strategy. Our goal, then, is to validate the added predictive value of the omics score in the male and in the female populations separately. The training set contains 88 female patients (54 events) and 74 male patients (51 events), while in the validation set there are 46 female patients (16 events) and 33 male patients (17 events). The sample sizes are very small, but not uncommonly so in studies dealing with omics data.

**Figure 9:**
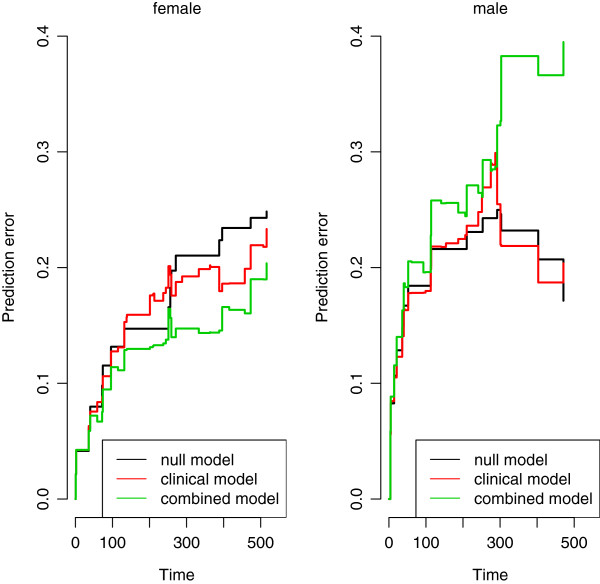
**AML: prediction error curves in female and male populations.** Acute myeloid leukemia: prediction error curves based on the Bier score computed in the validation set for the null (black line), the clinical (red line) and the combined (green line) models, fitted on the training data, for both the female (left) and the male (right) populations.

The results are striking: although the omics score was derived using the whole population, the difference in its usefulness in predicting the survival times for male and female patients is huge. While for the female subgroup its additional predictive value is sizable both in term of calibration (the calibration slope moves from 0.761 (sd =0.353) for the clinical model to 1.058 (sd =0.305) for the combined model) and discriminative ability (the C-index is equal to 0.632 for the clinical model and 0.689 for the combined model), in the male population the addition of the omics score worsens, in a very clear way, both the calibration (calibration slope from 0.698, sd =0.635, to 0.157, sd =0.397) and the discriminative ability (C-index from 0.584 to 0.493, even worse than the 0.500 representing the random situation) of the model. The prediction error curves plotted in Figure 9 clearly show the different effect of the omics score in the female and male populations: while the green curve (combined model) is definitely under the red one (clinical model) in the first graphic (female population), in the second graphic (male population) it is not only above the red curve, but also the black curve representing the prediction error curve of the null model.

**Figure 10:**
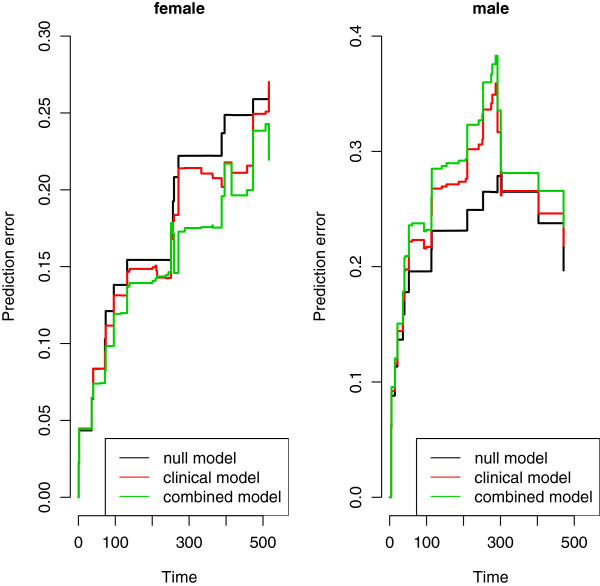
**AML: prediction error curves based on 10-fold cross-validation in female and male populations.** Acute myeloid leukemia: prediction error curves based on 10-fold cross-validation (100 replications) for the null (black lines), clinical (red lines) and combined (green lines) models in the female (left) and in the male (right) populations. Only the validation set is used.

To address the overfitting issue associated with this procedure, we then also repeat the analyses described above in both subgroups separately. Although both the positive effect (in the female subgroup) and the negative effect (in the male subgroup) of the omics score are substantially smaller in the validation set than in the training set in absolute value, the first impression is confirmed. The prediction error curves based on a 100 replication of a 10-fold cross-validation procedure (Figure 10) seem to confirm the results of the previous approaches (those performed in the two subgroups separately). The p-values from the combined model fitted on the validation set provide the same evidence, with a test on the nullity of the regression coefficient of the omics score yielding a p-value of 0.004 in the female population and 0.753 in the male one.

In particular, with regard to the overfitting issues, it is worth looking at the differences between the slopes of the prediction error curves in the graphics. If we look at Figure 9, we note that in the female population the prediction error curves for the three models have, more or less, the same slope, and the difference in their behaviors is basically a shift in the central part. The same happens in Figure 10. This is not the case for the male population. When the regression coefficients are estimated from the validation set (Figure 10), we experience a similar situation, but when the regression coefficients are estimated from the training set (Figure 9), the slope of the error prediction curve for the combined model has a completely different behavior. This could be the result of overfitting mechanisms that may affect the predictions in the male subgroup and not in the female subgroup. Nevertheless, the instability of the prediction error curves, derived by the small number of observations available in the two subgroups, does not allow us to draw any conclusion. The considerations made on the results of this subgroup analysis should be seen as an example of interpretation of the outcomes and not as an analysis on the specific dataset.

In any case, an unexpected relation between *sex* and the omics score seems to be present. A different way to investigate this relation consists of fitting a multivariate Cox model on the validation set, considering also the interaction between these two predictors. Although the p-value, as we stressed in the Methods section, is more related to the ability of the predictor to explain the outcome variability than to the predictive ability, its value for the interaction term (0.0499) seems to support the existence of an interaction. This result is hard to explain. Nothing in the medical literature seems to confirm such a strong interaction between sex and gene-expression for leukemia (there are only rare cases of specific gene deletions known to be related to sex, but they are not considered here). This is in contrast to the case, for example, of the interaction between the omics score and *FLT3-ITD*, which is well-known and was clearly stated in the original paper by Metzeler and colleagues [13]. This iteration could possibly be shown by performing the subgroup approach on the sample split between those patients with and those without the *FLT3-ITD*: unfortunately, the small number of patients without *FLT3-ITD* does not allow us to use this variable to illustrate the subgroup analysis. The total independence between *sex* and *FLT3-ITD* in the sample (if we test the hypothesis of independence through a Fisher exact test, we obtain a p-value equal to 1) allows us to exclude the presence of spurious correlation. Moreover, we note that in a multivariate Cox model which includes the interaction term score*sex, the effect of the omics score is more significant (p-value 0.0035) than in the model without the interaction term (p-value 0.031, see Table 4). If we consider the interaction FLT3-ITD*score in the Cox model, instead, the p-value of the omics score is high (0.4189), showing that all its explanatory ability lies in the interaction with *FLT3-ITD* (p-value = 0.0020). It is worth noting, however, that the effective sample size (in survival analysis we should consider relevant only those observations where an event occurs) in the subgroup analysis is small (16 events for women, 17 for men). The results may thus be affected by peculiar characteristics of the sample such as a specific pattern in the censoring scheme. To support this idea, we report the fact that the K-statistic computed in the two sub-populations (male and female) gives results completely different from the C-index: its value, indeed, is increased by the inclusion of the omics score in the prognostic index both in the female (from 0.684 for the clinical model to 0.694 for the combined model) and in the male (from 0.631 to 0.665) subgroups. We would like to stress that the provided interpretations should be understood as illustrative, and not as a conclusion for the leukemia study.

### Chronic lymphocytic leukemia

Here we show the possibilities to validate the added predictive value in a dataset where the training and validation data are different. We refer to the profile provided in Table 2 for a summary of the analyses performed.

**A. Evaluating the clinical model and the combined model on validation data.** The most notable peculiarity of this dataset is the different measurements of the gene expressions in the training and validation sets. Part of the advantage of the signature proposed in Herold et al. [19], indeed, lies in the relatively small number of involved genes (eight), which allows the practitioner to use a cheaper and more convenient platform to collect the data needed to compute the omics score. Nevertheless, the different measurements affect the validation strategy to be used for assessing the added predictive value of the omics score. In particular, it makes no sense to estimate a model which includes clinical predictors and omics score based on the training data and to apply this model to the validation data. Since the goal is to validate the added predictive value of the omics score when the gene expressions are collected with the technique used in the validation set, it is necessary to fit the considered models based on the validation data. This is what we do when applying the methods discussed below.

**B. Multivariate testing of the omics score in the validation data.** While it is not possible to compare the predictive ability of clinical and combined models fitted to the training set, methods fitting the coefficients of the models based on the validation set are fully applicable. In particular, a test can be conducted to test the nullity of the coefficient *β*_∗_ of the omics score in a multivariate model fitted on the validation set. The results presented in Table 6 (p-value <0.0001 for the omics score) confirm the utility of including the omics score in the predictive model for explaining the variability. We have already stressed that a significant p-value is not necessarily associated with added predictive ability and therefore we proceed with the cross-validation approach based on the Brier score.

**Table 6:** **Chronic lymphocytic leukemia: estimates of the log-hazard in a multivariate Cox model fitted on the validation data, with the standard deviations and the p-values related to the hypothesis of nullity of the coefficients (simple null hypothesis)**

Variable	Coeff	Sd (coeff)	P-value
*Omics score*	-0.589	0.150	8.65×10^-05^
*Age* (continuous)	0.113	0.023	6.82×10^-07^
*Sex* (female)	0.157	0.343	0.6472
*FISH* =1	0.171	0.459	0.7092
*FISH* =2	1.352	0.590	0.0219
*FISH* =3	-0.195	0.665	0.7694
*FISH* =4	-0.459	0.427	0.2823
*IGVH* (mutated)	0.695	0.416	0.0949

**Figure 11:**
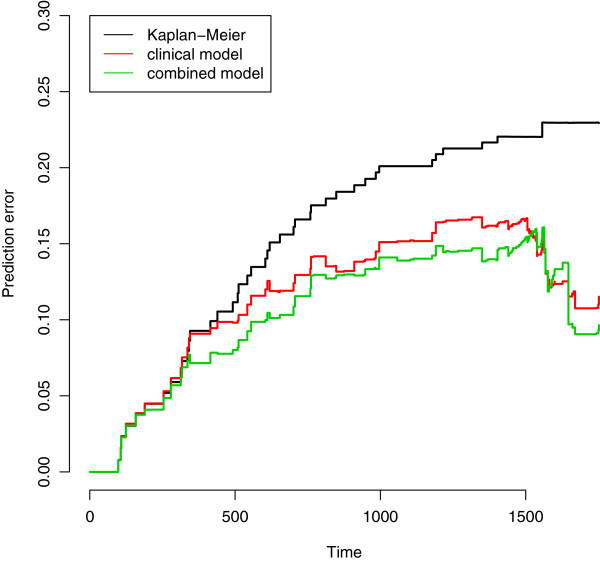
**CLL: prediction error curves based on 10-fold cross-validation in female and male populations.** Chronic lymphocytic leukemia: prediction error curves based on 10-fold cross-validation (100 replications).

**C. Comparison of the predictive accuracy of the models with and without omics score through cross-validation in the validation data.** We conduct the same analysis as for the AML dataset. Prediction error curves are displayed in Figure 11, clearly showing the added predictive value of the omics score. The curve of the combined model (green line) is clearly under the curve of the clinical model (red line). It can also be seen that the clinical model has better predictive ability than the null model (black line). These results are in line with the corresponding values of the integrated Brier score (null model: 0.142, clinical model: 0.113, combined model: 0.101, all computed up to 1500 days, value selected by looking at the Kaplan-Meier curves). We note that the prediction error curve for the combined model already starts to be below the one for the clinical and null models after only one year of follow-up, i.e., when the observations are numerous and the estimates stable. As in the previous example, these results are averaged over 100 repetitions of a 10-fold cross-validation procedure.

## Discussion

In this paper we deliberately focused on the case of the validation of omics scores fitted on training data in the context of survival analysis in the presence of a few clinical predictors. Other situations may be encountered in practice. Firstly, the omics score may be given from a previous study, in which case the overfitting issue leading to an overestimation of its effect is no longer relevant and the omics score can be treated as any other candidate biomarker. Secondly, there may be situations where a validation set is not available (typically because the available dataset is not large enough to be split). In this case, other (resampling-based) approaches may be taken to test predictive value and assess the gain of predictive accuracy [51, 52]. Thirdly, the outcome of interest may be something other than the survival time. Binary outcomes (e.g., responder vs. non-responder) are common. The evaluation criteria used to assess predictive accuracy are of course different in this case. Fourthly, one may also consider the added predictive value of a high-dimensional set of predictors versus another high-dimensional set of predictors. This situation is becoming more common with the multiplication of high-throughput technologies generating, for example, gene expression data, copy number variation data, or methylation data. Data integration is currently a hot topic in statistical bioinformatics and prediction methods handling this type of data are still in their infancy.

Furthermore, we did not address in our paper the problem of the construction of the omics score. We simply assumed that it was estimated based on the training data with an appropriate method. The construction of such an omics score is of course not trivial and has indeed been the subject of numerous publications in biostatistics and bioinformatics in the last decade. From the point of view of predictive accuracy it may be advantageous to construct the omics score while taking the clinical predictors into account [47, 53, 54] in order to focus on the residual variability, a fact that we did not consider in this paper but plan to investigate in a subsequent study. The two omics scores analyzed here, indeed, were constructed without this expedient, and optimized to take the place of the clinical predictors rather than focusing on the added predictive value of the omics data.

Finally, we point out that, even in the case considered in our paper (validation of omics scores fitted on training data in the context of survival analysis in the presence of a few clinical predictors), further approaches are conceivable. For example, other evaluation criteria for prediction models may be considered; see [23] for a recent overview in the context of external validation. When considering combined prediction models we focused on the multivariate Cox model with clinical predictors and omics score as covariates and with linear effects only. Of course further methods could be considered in place of the Cox model with linear effects, including models with time-varying coefficients, parametric models or non-linear transformations of the predictors such as fractional polynomials.

As soon as one “tries out” many procedures for assessing added predictive value, however, there is a risk of conscious or subconscious “fishing for significance” – in this case “fishing for added predictive value”. To avoid such pitfalls, it is important that the choice of the method used in the final analyses presented in the paper is not driven by the significance of its results. If several sensible analysis strategies are adopted successively by the data analysts, they should consider reporting all results, not just the most impressive in terms of added predictive value.

Here we have summarized all our analyses in REMARK type profile tables (namely, Tables 1 and 2), in order to increase transparency and to allow the reader to easily go through the study. Transparency is an important issues, and was also highlighted in the US National Cancer Institute’s criteria for the clinical applicability of an omics-based predictor [7, 17]. Among the 30 points listed in this checklist, one is clearly devoted to the validation of the omics-based predictor: the validation should be analytically and statistically rigorous. These papers also stress the importance of reproducibility of the analysis: in this vein, we provide all R-codes used to obtain the results presented in this paper at http://www.ibe.med.uni-muenchen.de/organisation/mitarbeiter/070_drittmittel/de_bin/index.html.

## Conclusion

In this paper we illustrated and critically discussed the application of various methods with the aim of assessing the added predictive value of omics scores through the use of a validation set. In a nutshell, our study based on two recent leukemia datasets outlined that:

-  When testing is performed for a multivariate model on the validation data, the omics score may have a significant p-value but show poor or no added predictive value when measured using criteria such as the Brier score. This is because a test in multivariate regression tests whether the effect of the omics score is zero but does not assess how much accuracy can be gained through its inclusion in the model.
-  To gain information on – and “validate” – predictive value, it is necessary to apply models with and without the omics score to the validation data. There are essentially two ways to do that.
-  The first approach (denoted “Evaluating the clinical model and the combined model on validation data” in this paper) consists of fitting a clinical model and a combined model on the training data and comparing the prediction accuracy of both models on the validation data. This is essentially the most intuitive way to proceed in low-dimensional settings. The problem in high-dimensional settings is that the omics score is likely to overfit the training data. As a result, its effect might be overestimated when its regression coefficient is estimated using again the same set using for its construction. We have seen how this leads to serious problems, especially in term of bad calibration. Furthermore, this approach is not applicable when the omics data has been measured with different techniques in the training and validation sets, as in the CLL data.
-  The second approach, which we recommend in high-dimensional settings, consists of using a cross-validation-like procedure to compare models with and without the omics score using the validation set. By using the validation set only, we avoid the overfitting problem described above. When using this approach, it is recommended performing as many repetitions of CV as computationally feasible (and to average the results over the repetitions) in order to achieve more stable results.
-  Alternatively, one could also fit the models on the validation set and use an additional third set to assess them. This approach would avoid the use of cross-validation procedures that are known to be affected by a high variance, especially in high-dimensional settings. However, the opportunity to assess the models based on a third set is rarely given in the context of omics data, since datasets are usually too small to be split.
-  In any case, it is important that training and validation sets are completely independent. The practice of evaluating the prediction ability of a model, correctly fitted only on the training set, on the whole dataset obtained by merging the training and validation sets is not appropriate. This would indeed result in an overoptimistic estimation of prediction accuracy, because of the overoptimism observed due to the evaluation on the training data, only partially mitigated by the correct estimate obtained on the independent validation data [7, 55].
-  All in all, our procedures are in line with the recommendations given in a recent paper by Pepe and colleagues [22]. This paper suggests that, in the case of binary outcome, all the tests based on the equality between the discriminative abilities of the clinical and the combined scores refer to the same null hypothesis, namely the nullity of the coefficient of a predictor in a regression model. Assuming that this statement also roughly applies to the survival analysis framework considered in our paper, it would mean that we can rely on the likelihood test performed on the regression coefficient of the omics score in the combined Cox model to *test* the difference in performance of the models with and without omics predictors. However, the same authors also claim that estimating the magnitude of the improvement in the prediction ability is much more important than testing its presence [22]. This cannot be done by looking at the regression coefficient of the omics score, as often discussed in the literature [56, 57] and illustrated through our AML data example. In this paper we have seen some procedures to quantify the improvement in prediction accuracy of a model containing an omics score derived from high-dimensional data, in order to validate its added predictive value.
-  Subgroup analyses might give valuable insights into the predictive value of the score, and therefore illustrated through the example of the AML dataset. Normally, the subgroups analysis should be inspired by a clear biological reason and, importantly, performed as far as allowed by the sample sizes. However, one should keep in mind that these analyses are possibly affected by multiple testing issues. Their results should be considered from an explorative perspective.

Due to our experience with the analysis of the two considered leukemia datasets and further similar datasets (data not shown), we recommend comparing the predictive accuracy of the models with and without omics score through a resampling-based approach on the validation data. The repeated cross-validation procedure is the natural candidate, but we have seen that alternative methods can be implemented.
